# Neuronal P2X7 receptor-induced reactive oxygen species production contributes to nociceptive behavior in mice

**DOI:** 10.1038/s41598-017-03813-7

**Published:** 2017-06-14

**Authors:** Frances M. Munoz, Ruby Gao, Yuzhen Tian, Brian A. Henstenburg, James E. Barrett, Huijuan Hu

**Affiliations:** 1 Department of Pharmacology and Physiology, Drexel University College of Medicine, Philadelphia, USA; 2 Department of Neurobiology and Anatomy, Drexel University College of Medicine, Philadelphia, USA

## Abstract

ATP can activate a variety of pathways through P2 purinoreceptors, leading to neuroprotection and pathology in the CNS. Among all P2X receptors, the P2X7 receptor (P2X7R) is a well-defined therapeutic target for inflammatory and neuropathic pain. Activation of P2X7R can generate reactive oxygen species (ROS) in macrophages and microglia. However, the role of ROS in P2X7R–induced pain remains un﻿explored. Here, we investigated the downstream effects of neuronal P2X7R activation in the spinal cord. We found that ATP induces ROS production in spinal cord dorsal horn neurons, an effect eliminated by ROS scavenger N-tert-butyl-α-phenylnitrone (PBN) and P2X7R antagonist A438079. A similar effect was observed with a P2X7R agonist, BzATP, and was attenuated by a NADPH oxidase inhibitor apocynin. Intrathecal administration of BzATP resulted in ROS production in the spinal cord and oxidative DNA damage in dorsal horn neurons. BzATP also induced robust biphasic spontaneous nociceptive behavior. Pre-treatment with A438079 abolished all BzATP-induced nociceptive behaviors, while ROS scavengers dose-dependently attenuated the secondary response. Here, we provide evidence that neuronal P2X7R activation leads to ROS production and subsequent nociceptive pain in mice. Together, the data indicate that P2X7R-induced ROS play a critical role in the P2X7R signaling pathway of the CNS.

## Introduction

Adenosine triphosphate (ATP) is a ubiquitously abundant signaling molecule that can activate a vast variety of pathways, allowing downstream effects that can lead to both neuroprotection and pathology in the central nervous system (CNS). Non-neuronal cells and neurons release ATP in order to communicate with each other and other types of cells in the CNS by activating P2 purinoreceptors^1, 2^. These receptors are sub classified as ionotropic P2XRs and metabotropic P2YRs, and are broadly distributed in neurons and glial cells^2^. While activation of P2YRs is mainly coupled to phospholipase C (PLC), activation of the ionotropic P2XRs cause the opening of cation permeable channels, initiating intracellular Ca^2+^ mobilization and further downstream signaling^3, 4^. Among all P2X receptors, the P2X7 receptor (P2X7R) is a well-defined therapeutic target for inflammatory diseases^5^, and has been found in the central and peripheral nervous systems^6–8^. Specifically, P2X7R is known to be involved in cell proliferation, apoptosis, modulation of neurotransmitter release, and microglial and astrocyte activation in the nervous system^9, 10^. Activation of P2X7R with a selective agonist, 2′(3′)-O-(4-Benzoylbenzoyl) adenosine-5′-triphosphate tri (triethylammonium) salt (BzATP), can also increase the production of proinflammatory cytokines from both macrophages and microglia^11^. Despite controversy in the field over the role of neuronal P2X7R, recent findings report functional expression of P2X7Rs in neurons^12–15^. Moreover, activation of P2X7R in neurons can lead to cell death^14, 15^.

Reactive oxygen species (ROS) production can occur as a natural byproduct of cellular metabolism, and plays a role both in intracellular and extracellular signaling. Physiologically, generation of ROS occurs in subcellular compartments such as the mitochondria, the endoplasmic reticulum (ER), peroxisomes, and on the plasma membrane through NAPDH oxidases and lipoxygenases^16^. Under pathological conditions, cells can accumulate high amounts of ROS, which react with proteins, lipids, carbohydrates, and nucleic acids causing irreversible changes that subsequently lead to cellular damage^17^. An excessive and/or sustained increase in ROS production has been implicated in the pathogenesis of many diseases including atherosclerosis, rheumatoid arthritis, and diseases in the CNS such as Parkinson’s disease, Alzheimer’s disease and chronic persistent pain^18^. Early studies reported that many forms of ROS can mediate inflammatory pain and contribute to pain behaviors associated with inflammation and neuropathic injury^19–21^. Moreover, recent studies have demonstrated that ROS play an important role in both neuropathic pain and inflammatory pain in rats, specifically through interactions with NMDA receptors^22, 23^. Despite growing knowledge of ROS in pain, however, little is known regarding the role of ROS in acute nociception.

Recent studies have shown that P2X7R activation can generate ROS in macrophages and microglia following stimulation with ATP or BzATP, an effect that can be blocked by P2X7R inhibitors such as oxidized ATP (oATP) and Brilliant Blue G (BBG)^24–26^. P2X7R activation in microglia can also lead to generation of ROS, an increase in proinflammatory cytokines, and subsequent brain injury^27^. In addition, NADPH oxidase (NOX) plays a significant role in P2X7R-induced ROS production in macrophages and microglia^28, 29^. Thus, generation of ROS following P2X7R activation is well established in other cell types. ATP is one of the major mediators of pain signaling in the spinal cord^30^, and P2X7R can modulate behavioral responses to pain^31^. Interestingly, in mice lacking P2X7R, inflammatory pain and neuropathic pain are completely abolished^32^. Moreover, P2X7R has proven to be an important therapeutic target in inflammatory pain and neuropathic pain^5, 33^. However, the mechanisms by which P2X7R contributes to pain remain poorly understood, and the role of ROS in P2X7R–induced pain has yet to be explored. In the current study, we sought to determine the consequence of P2X7R activation in dorsal horn neurons. Here, we provide evidence that neuronal P2X7R activation leads to ROS production and subsequent nociceptive pain in mice.

## Results

### P2X7R activation increases ROS in spinal cord dorsal horn neurons

**Figure 1:**
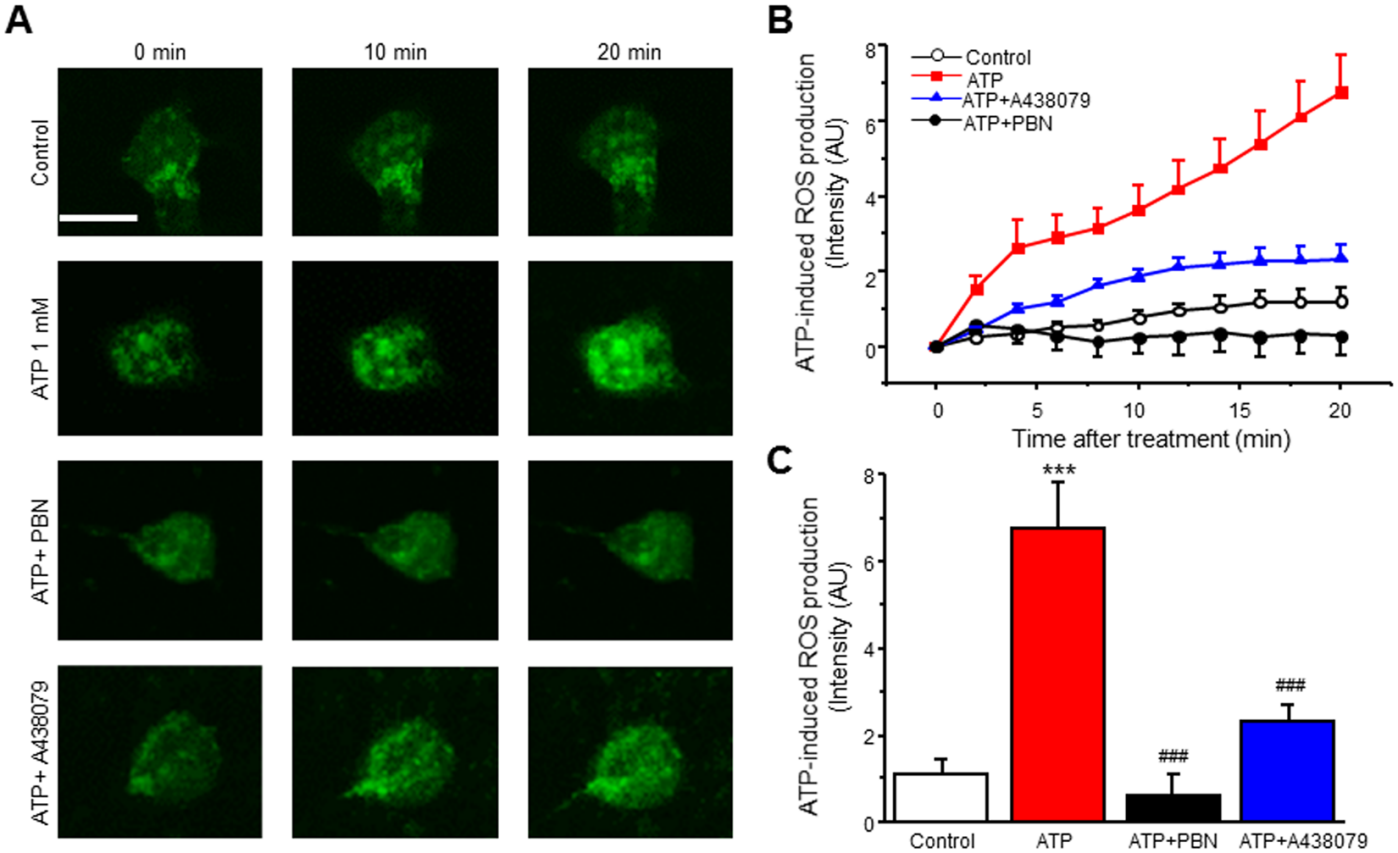
ATP induces ROS increase in spinal cord dorsal horn (SCDH) neurons. (**A**) Representative of live cell confocal images captured before (0 min) and after application of 1 mM ATP in the presence of vehicle, PBN or A438079. (**B**) Time lapse of ROS production after ATP (1 mM) administration in the presence of vehicle, PBN or A438079. (**C**) Summary of the effects of PBN or A438079 on ATP-induced ROS production. Values represent mean ± SEM, n = 11–12 neurons. ***P < 0.01 compared to control, ^###^ *P* < 0.001 compared to ATP group by the one-way ANOVA. Scale bar 10 µM.

Previous studies have shown P2X7R activation can induce ROS production in microglia. To determine whether ROS increases following P2X7R activation in cultured spinal cord dorsal horn (SCDH) neurons, cells were loaded with CellROX Green, a fluorescent ROS indicator, and subjected to live cell confocal microscopy. Early reports show that P2X7R activation in astrocytes can lead to glutamate release^34, 35^. In order to eliminate glutamate release that may subsequently activate neuronal ROS increases in our cultures, cells were loaded with D-AP5 (30 µM) and CNQX (10 µM), NMDA and AMPA receptor antagonists respectively, 10 min prior to imaging. Following drug administration, images were taken at 2 min intervals for 20 minutes. ATP (1 mM), which activates the P2 purinoreceptor family, increased ROS in a time-dependent manner (Fig. 1A,B). To confirm CellROX intensity increased due to ROS production, cells were pre-treated with the ROS scavenger N-tert-butyl-α-phenylnitrone (PBN, 30 µM) for 30 min. Co-treatment of ATP and PBN showed a complete inhibition of ROS production (Fig. 1B,C). To determine whether ROS increases were occurring due to P2X7R activation, neurons were pre-treated for 10 min with a competitive P2X7R antagonist, A438079, then co-treated with ATP. Interestingly, inhibition with A438079 (10 µM) partially attenuated ROS production in SCDH neurons (Fig. 1B,C).

**Figure 2:**
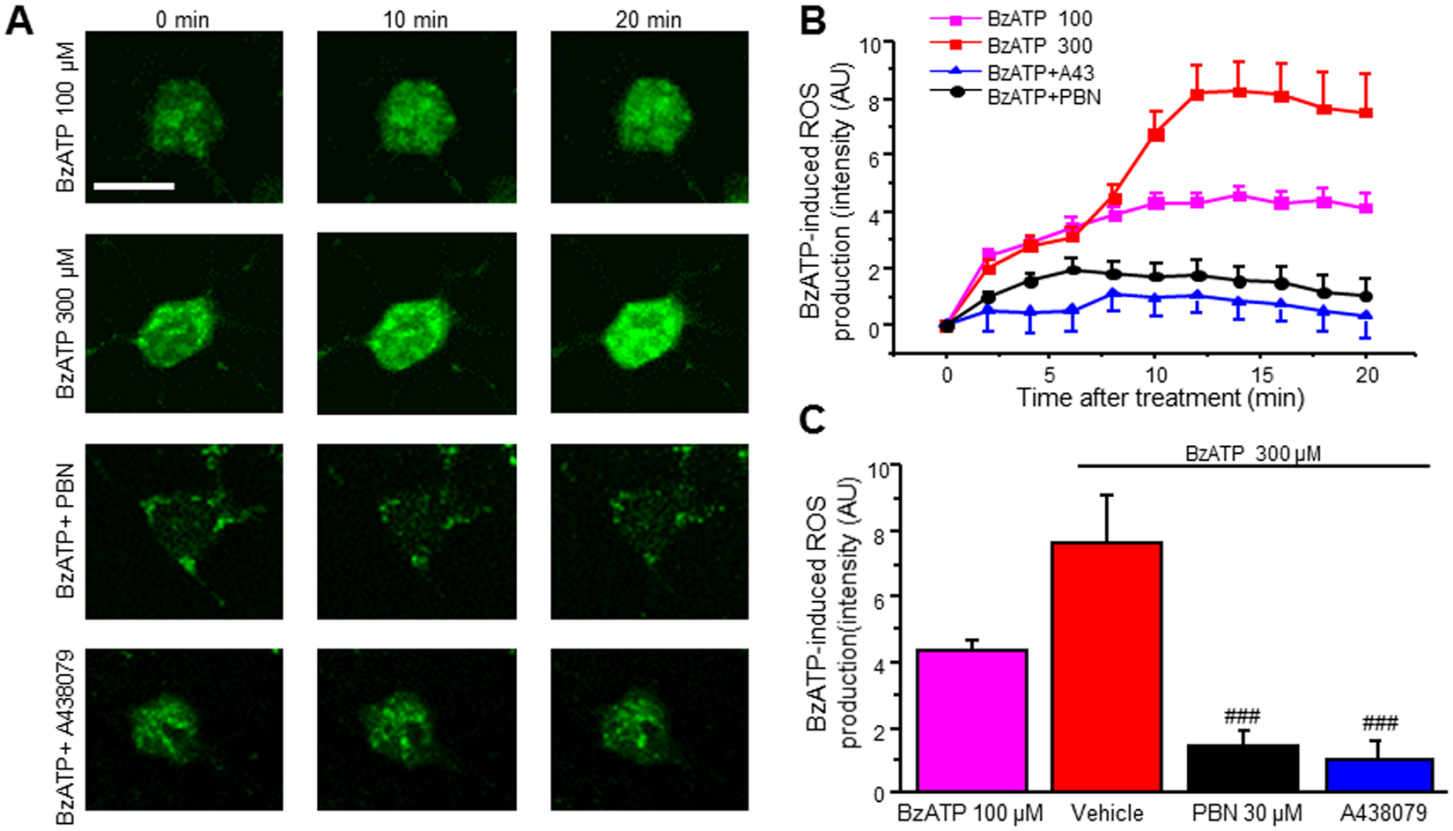
P2X7R activation with BzATP induces ROS production in SCDH neurons. (**A**) Representative of live cell confocal images captured before (0 min) and after application of BzATP in the presence of vehicle, PBN or A438079. (**B**) Time lapse of ROS production after BzATP (300 μM) administration in the presence of vehicle, PBN or A438079. (**C**) Summary of the effects of PBN or A438079 on BzATP-induced ROS production. Values represent mean ± SEM, n = 10 –19 neurons. ^###^ *P* < 0.01 compared to BzATP group by the one-way ANOVA. Scale bar 10 µm.

To further confirm P2X7R activation increases ROS in SCDH neurons, we employed a P2X7R agonist, 2′(3′)-O-(4-Benzoylbenzoyl)adenosine-5′-triphosphate tri(triethylammonium) salt (BzATP). BzATP induced a robust increase in a dose- and time-dependent manner, reaching maximal ROS levels around 12 min (Fig. 2A,B). About 79% of neurons showed ROS increase to BzATP (300 µM) treatment after 20 min (33 of 42 neurons). The effect was abolished by pre-treatment with ROS scavenger PBN (Fig. 2B,C). When we pre-treated neurons with A438079 (A43) and co-treated with BzATP (300 µM), ROS production was completely blocked (Fig. 2B,C). Together, our results suggest that P2X7R activation induces ROS production in SCDH neurons.

### P2X7R activation may increase ROS through NAPDH oxidase

**Figure 3:**
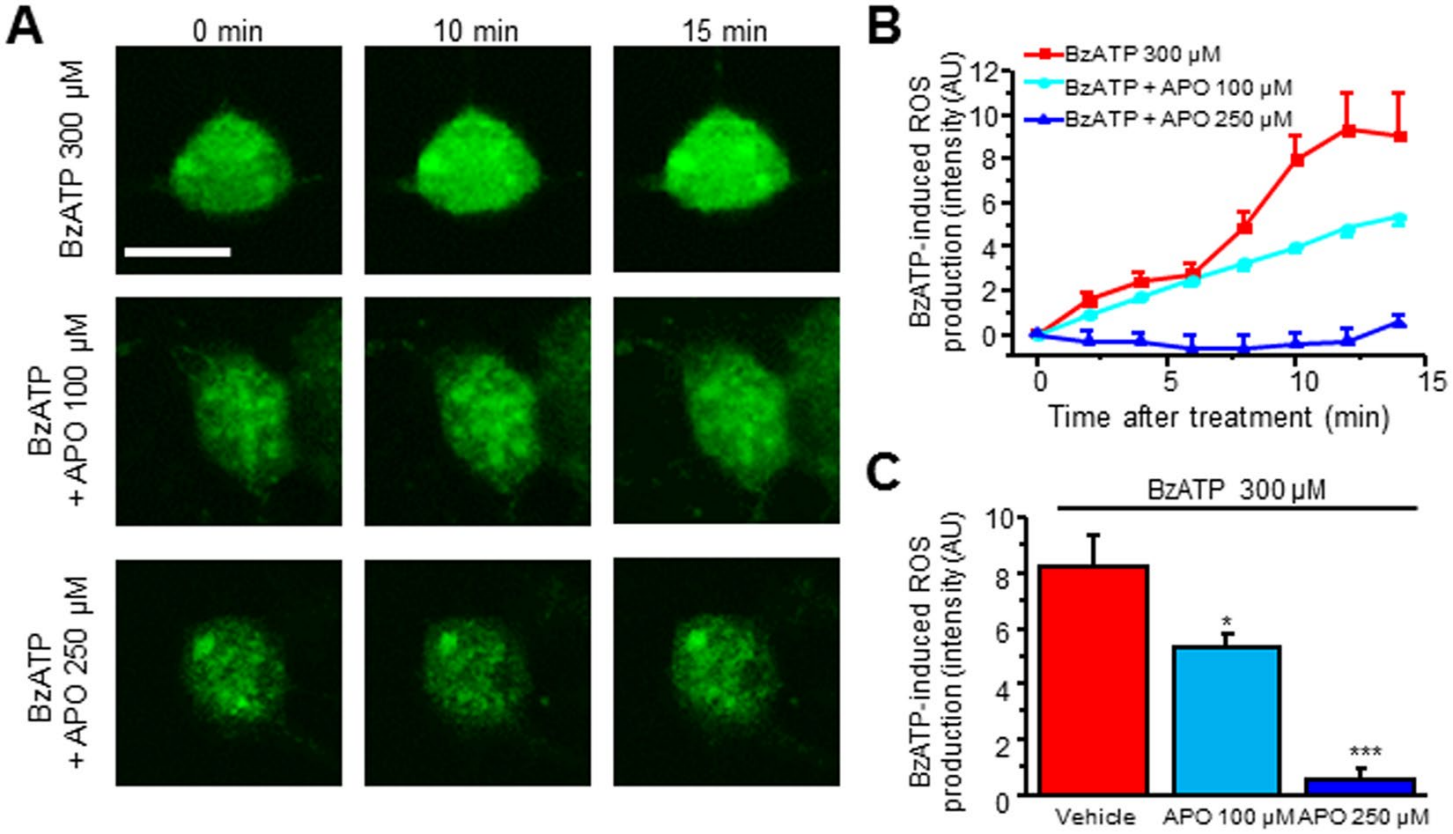
NOX inhibition attenuates BzATP-mediated ROS production. (**A**) Representative of live cell confocal images captured before (0 min) and after application of BzATP in the presence of vehicle or apocynin (100 µM, 250 µM). (**B**) Time lapse of ROS production after BzATP (300 μM) administration in the presence of vehicle or apocynin. (**C**) Summary of the effects of apocynin on BzATP-induced ROS production. Values represent mean ± SEM, n = 6–12 neurons. *P < 0.05, ***P < 0.001 compared to BzATP group by the one-way ANOVA. Scale bar 10 µm.

Previous studies have demonstrated P2X7R activation promotes NOX-mediated ROS production in microglia^29, 36^. Therefore, we sought to determine whether NADPH oxidase (NOX) contributed to P2X7R-mediated ROS increases in SCDH neurons. Similar to our previous experiments, neurons were loaded with CellROX green and subjected to live cell confocal microscopy. Prior to imaging, neurons were pre-treated with a NOX inhibitor apocynin. Indeed, pre-treatment with apocynin (APO, 100 µM) partially reduced BzATP-mediated ROS production when compared to BzATP (300 µM) alone (Fig. 3A,B). We therefore increased the apocynin concentration to 250 µM, which strongly reduces P2X7R-mediated ROS production in microglia^37^. Apocynin 250 µM abolished P2X7R-mediated increases (Fig. 3B,C).

### PBN does not affect P2X7R function

**Figure 4:**
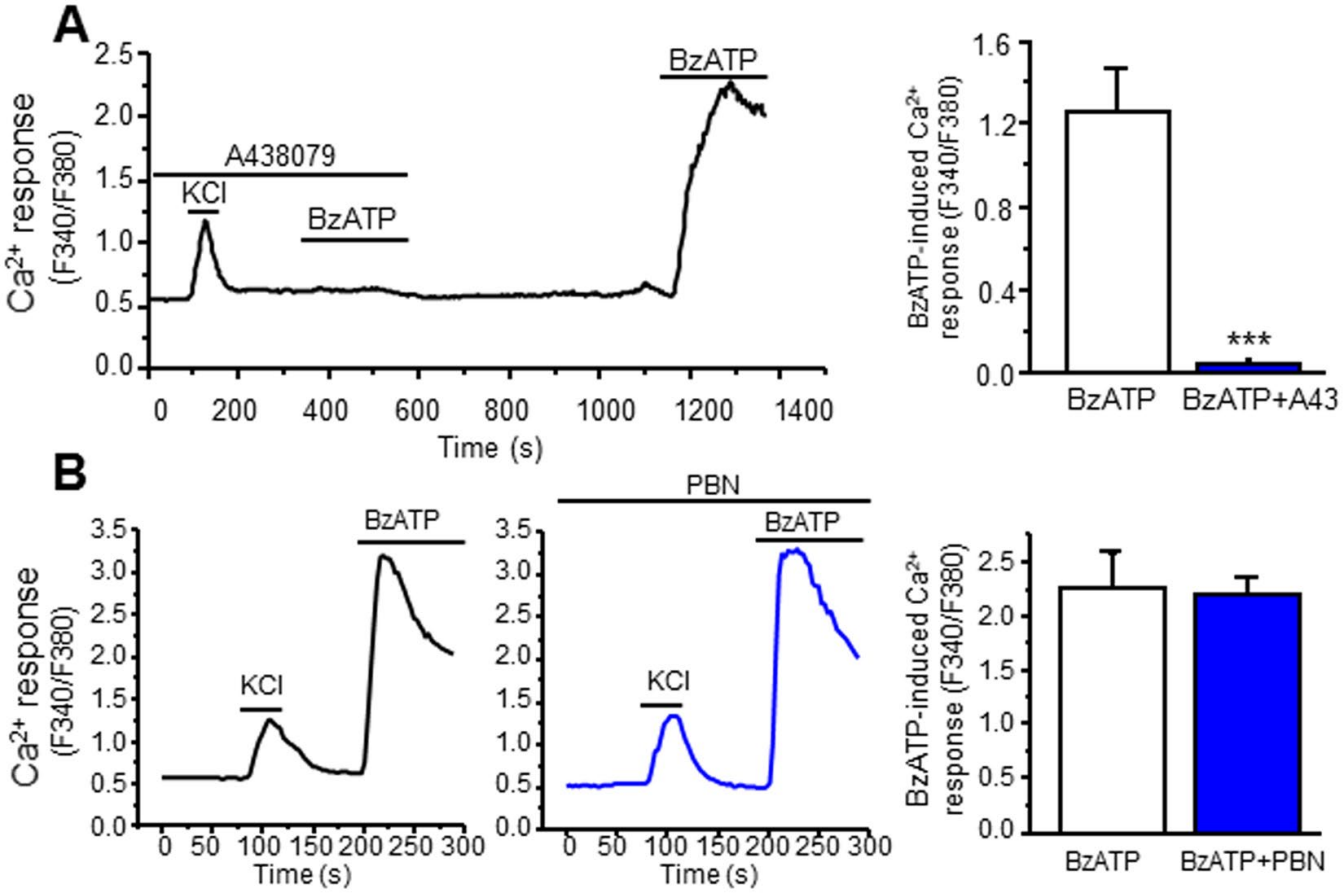
PBN does not affect P2X7R-induced Ca^2+^ increases. (**A**) BzATP induces intracellular Ca^2+^ increases through P2X7R activation in SCDH neurons. Left panel: Representative Ca^2+^ imaging recording of KCl- and BzATP-induced Ca^2+^ increases in the presence and absence of A438079. Right panel: Summary of the effects of A438079 on BzATP-induced calcium response. **(** **B** **)**﻿ PBN (30 µM) does not affect P2X7R function. Left panel: Representative Ca^2+^ imaging recordings of BzATP-induced Ca^2+^ increases in the presence and absence of PBN. Right panel: Summary of the effects of PBN on BzATP-induced calcium response. Values represent mean ± SEM, n = 15–16 neurons. ***P < 0.001 when compared to BzATP 300 µM by the unpaired Student’s *t* test.

To determine whether ROS scavenger PBN reduced ROS production by affecting the function of P2X7R, we performed calcium imaging experiments. SCDH neurons were loaded with the Ca^2+^ dye Fura-2AM in HBSS. Under the microscope, neurons were clearly distinguishable from glia. They appeared bright, and had small, smooth somata and visible processes. 60 mM KCl, which produces a fast and transient Ca^2+^ response in neurons, was also used to further determine neurons in our culture. First, we confirmed P2X7R activation by BzATP in neurons. About 75% of neurons responded positively to BzATP treatment (27 of 36 neurons). Pre-treatment with P2X7R antagonist, A438079 (10 µM), completely blocked intracellular Ca^2+^ responses caused by BzATP (300 µM) (Fig. 4A). Neurons were then washed with 2 mM Ca^2+^ Tyrode’s solution for 10 minutes and stimulated with BzATP once more. Following the wash, BzATP induced significant intracellular Ca^2+^ increases (Fig. 4A). The data indicate BzATP activates P2X7R in SCDH neurons.

Next, we pre-treated neurons with PBN (30 µM) in 2 mM Ca^2+^ Tyrode’s solution for 30 minutes prior to calcium imaging. Pre-treatment and co-treatment with PBN did not affect P2X7R activation by BzATP (Fig. 4B). Thus, PBN reduces ROS increases through its scavenging properties.

### Activation of P2X7R with BzATP increases ROS in the spinal cord dorsal horn of mice

**Figure 5:**
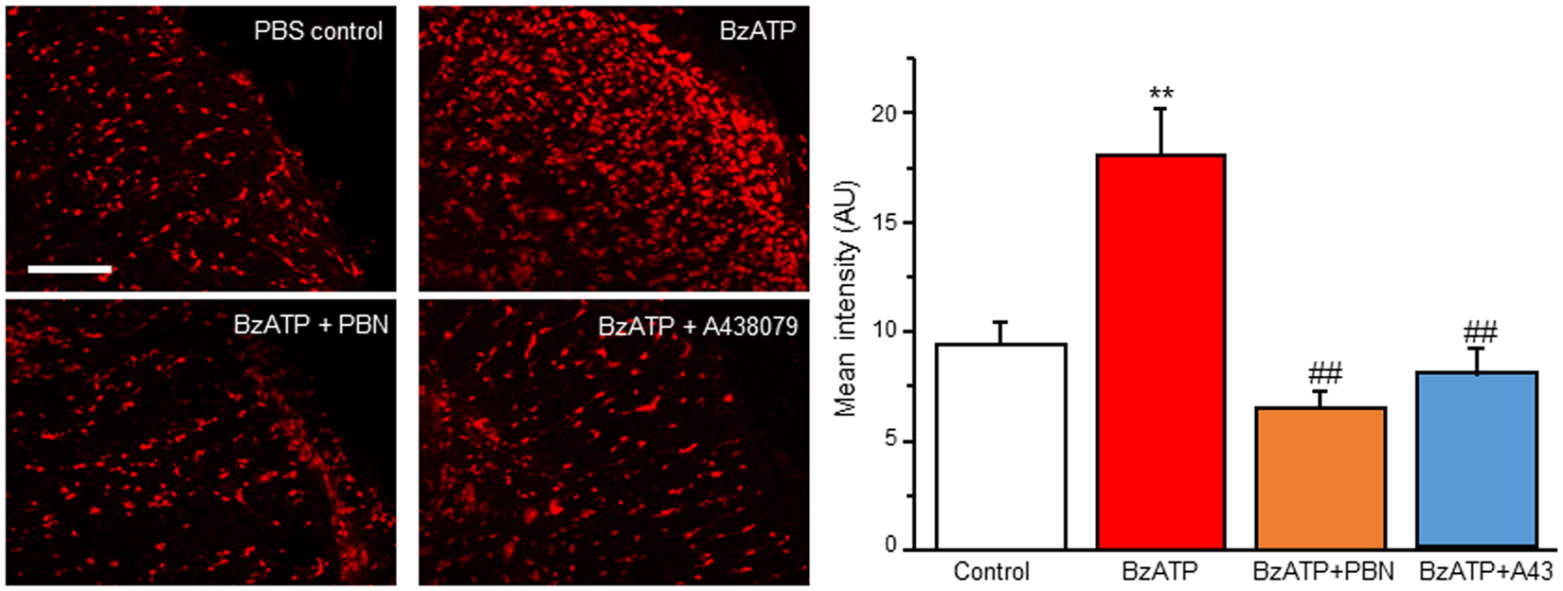
BzATP increases ROS in the spinal cord dorsal horn *in vivo*. Left panel: Confocal images of BzATP-induced ROS production in the presence and absence of PBN and A438079. Right panel: Summary of the effects of A438079 and PBN on BzATP-induced ROS production. Values represent mean ± SEM, n = 3–6. **P < 0.01 when compared to control, ^##^ *P* < 0.01 when compared to BzATP by the one-way ANOVA. Scale bar 100 µm.

To determine whether P2X7R activation could also induce ROS production *in vivo*, the superoxide indicator dihydroethidium (DHE, 1 µM) was used, and intensity was measured in the dorsal horn of the L4-L5 spinal segment of mice. Mice were administered with a single intrathecal injection of BzATP (30 nmol). After 10 min (according to peak ROS levels in cultured dorsal horn neurons), the spinal cord was removed, frozen, sectioned, and stained using DHE, then viewed under a confocal microscope. Mice intrathecally injected with saline showed basal levels of ROS in the SCDH, while intrathecal administration of BzATP showed a robust increase in ROS production (Fig. 5). Additionally, mice pre-treated with PBN (100 mg/kg, i.p.) for 30 min prior to the intrathecal injection of BzATP showed a complete reduction in DHE intensity levels in the dorsal horn. Pre-treatment with A438079 (100 mg/kg, i.p.) for 30 min prior to the injection also completely reduced DHE levels in the dorsal horn, thus suggesting ROS increases induced by BzATP occur through P2X7R. These results indicate that P2X7R activation increases ROS production in the SCDH of mice.

### BzATP induces oxidative damage in spinal cord neurons

**Figure 6:**
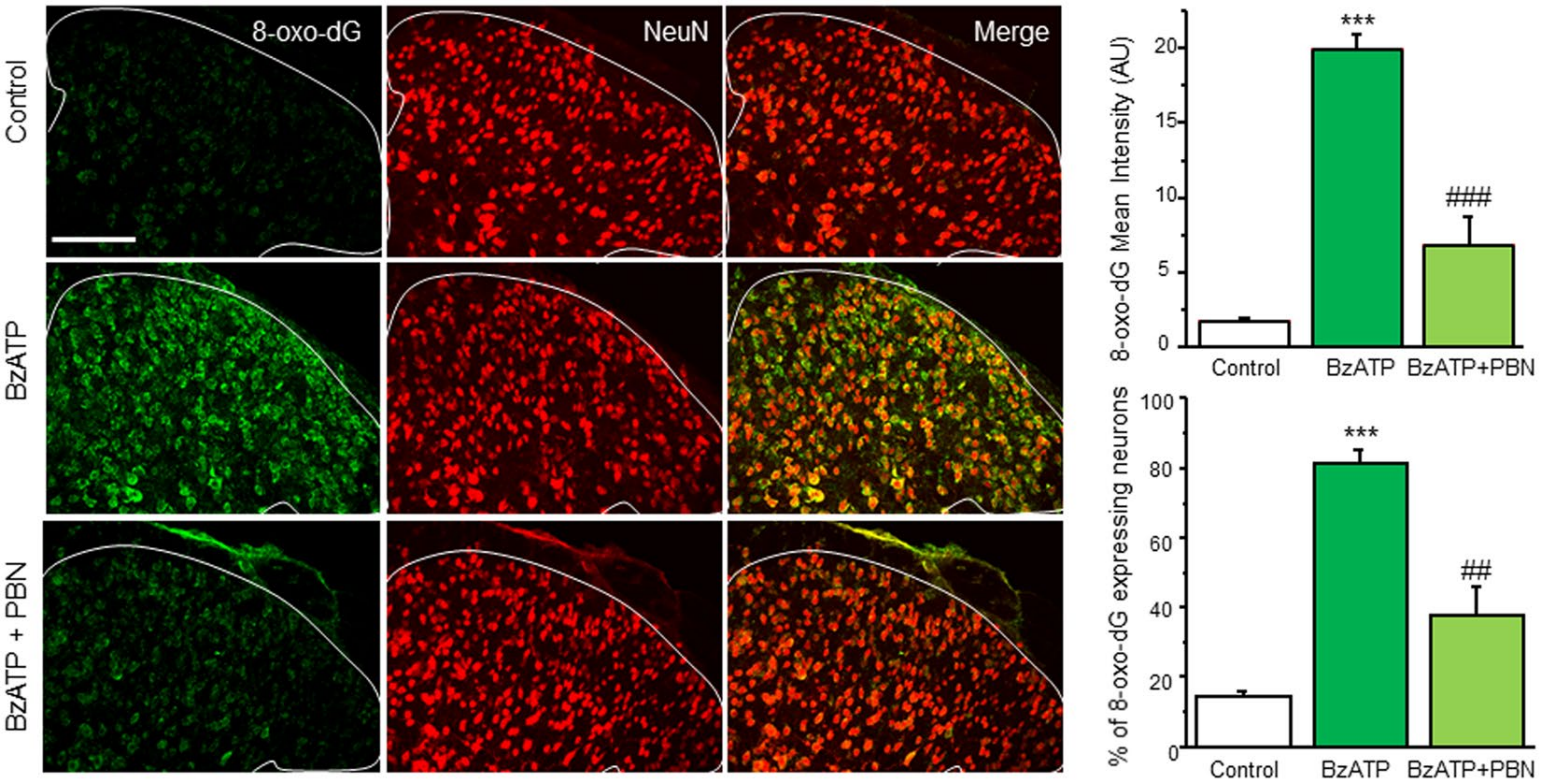
BzATP increases 8-oxo-dG in spinal cord dorsal horn neurons *in vivo*. Left panel: Confocal images of BzATP-induced oxidative DNA damage as marked by 8-oxo-dG (green) with and without PBN pre-treatment. Spinal cords were double stained with NeuN, a neuronal marker (red). Top right panel: Summary of 8-oxo-dG intensity in BzATP-induced oxidative DNA damage. Bottom right panel: Percent colocalization of 8-oxo-dG expressing neurons. Values represent mean ± SEM, n = 3–5. ***P < 0.001 when compared to control, ^##^P < 0.01, ^###^ *P* < 0.001 when compared to BzATP by the one-way ANOVA. Scale bar 100 µm.

Aberrant ROS can react with DNA to form various oxidized bases and nucleotides. One of the most prevalent oxidized bases in DNA is 8-oxo-2′-deoxyguanosine (8-oxo-dG), which causes a faulty pairing with adenosine on the opposite template by many DNA polymerases, and ultimately leads to cellular damage and, in higher amounts, cell death^38, 39^. Consequently, 8-oxo-dG is used as a constituent marker for oxidative DNA damage^40, 41^. To confirm whether P2X7R activation can increase ROS in neurons, mice were given intrathecal BzATP as described above. Animals were perfused, and the L4-L5 area of the spinal cords were removed, cryoprotected in sucrose, sliced then incubated with primary antibodies against 8-oxo-dG and NeuN, a marker for neurons. Very little 8-oxo-dG was detected in the SCDH of PBS controls. However, slices from mice that were given an intrathecal injection of BzATP showed a marked increase in 8-oxo-dG in the SCDH when compared to their saline controls (Fig. 6). Similar to our DHE staining (Fig. 5), pre-treatment with PBN significantly reduced 8-oxo-dG levels in the SCDH (Fig. 6). Interestingly, 81% of total NeuN colocalized with 8-oxo-dG, suggesting an increase in ROS production occurs mainly in spinal cord dorsal horn neurons.

### Acute administration of BzATP does not induce microglial and astrocyte activation

Previous studies have shown that P2X7R activation can cause microglial activation^42^. Moreover, intrathecal administration with ATP induces microglial and astrocyte activation as early as 1 day and up to 3 days^43^. To determine whether microglia or astrocytes were activated in BzATP-induced acute pain, mice were given an intrathecal injection of either PBS or BzATP (30 nmol) for 10 min, and spinal cords were collected as described in the previous section. Spinal cord sections were then incubated with the primary antibodies against the microglial activation marker Iba1 or against the astrocytic activation marker GFAP. Some Iba1 positive cells were observed in the dorsal horn of spinal cord sections from both PBS- and BzATP-treated mice, while GFAP positive cells were observed in the lamina I of the dorsal horn (Supplemental Figure 1). However, there were no significant differences in Iba1 and GFAP staining between PBS and BzATP-injected animals (Supplemental Figure 1). Moreover, Iba1 and GFAP positive cells do not appear branched and had no extensive processes indicative of microglial or astrocytic activation. Thus, our results suggest microglial and astrocytic activation do not occur after acute administration of BzATP.

### BzATP induces spontaneous nociceptive behavior

**Figure 7:**
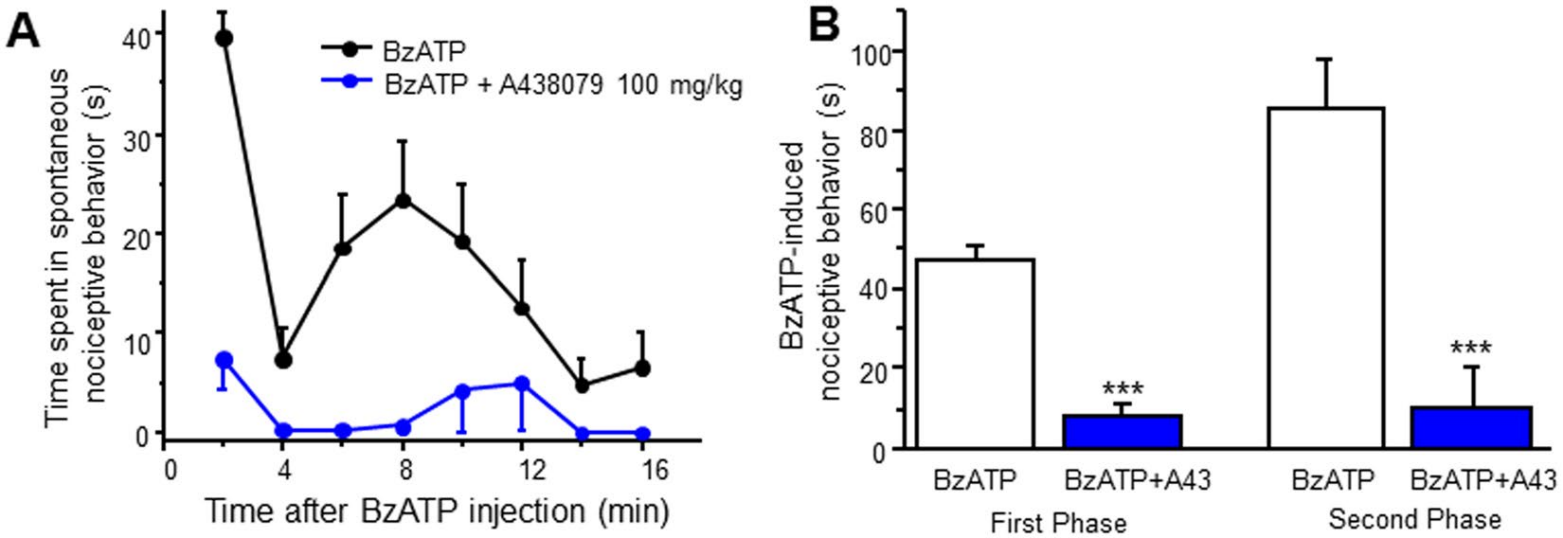
BzATP induces spontaneous nociceptive behavior. (**A**) Time course of spontaneous nociceptive behavior following an intrathecal injection of BzATP (30 nmol) with and without A438079 pre-treatment. Spontaneous nociceptive behavior was measured every 2 min for 16 min. (**B**) Total time spent in spontaneous nociceptive behavior. First phase of behavior includes time 0–4 min. Second phase includes 4–16 min. Values represent mean ± SEM, n = 5–8. ***P < 0.001 when compared to BzATP by the Student’s *t* test.

Previous studies show P2X7R antagonists reduce pain associated with spinal cord injury and long term potentiation, suggesting P2X7Rs may play a central role in pain^12, 44^. We therefore tested whether BzATP could induce spontaneous nociceptive behavior in mice, which includes caudally oriented licking of the flanks, tail, and hind paws. Mice were habituated individually in a Plexiglas cage for 30 min, and were then administered a single intrathecal injection of BzATP. Time spent in nociceptive behavior was recorded every 2 min for 16 min. No spontaneous nociceptive behavior was exhibited with intrathecal administration of PBS. Intrathecal injection of BzATP induced robust spontaneous nociceptive responses. Mice demonstrated an immediate first response within the first 2 min followed by a secondary spontaneous nociceptive response lasting about 16 min (Fig. 7A). A 30 min pre-treatment with the P2X7R antagonist A438079 (100 mg/kg, i.p.) completely abolished both the first response and second response (Fig. 7A,B). Together, our results indicate that P2X7R activation can induce spontaneous nociceptive behavior.

### ROS scavengers attenuate BzATP-induced nociceptive behavior

**Figure 8:**
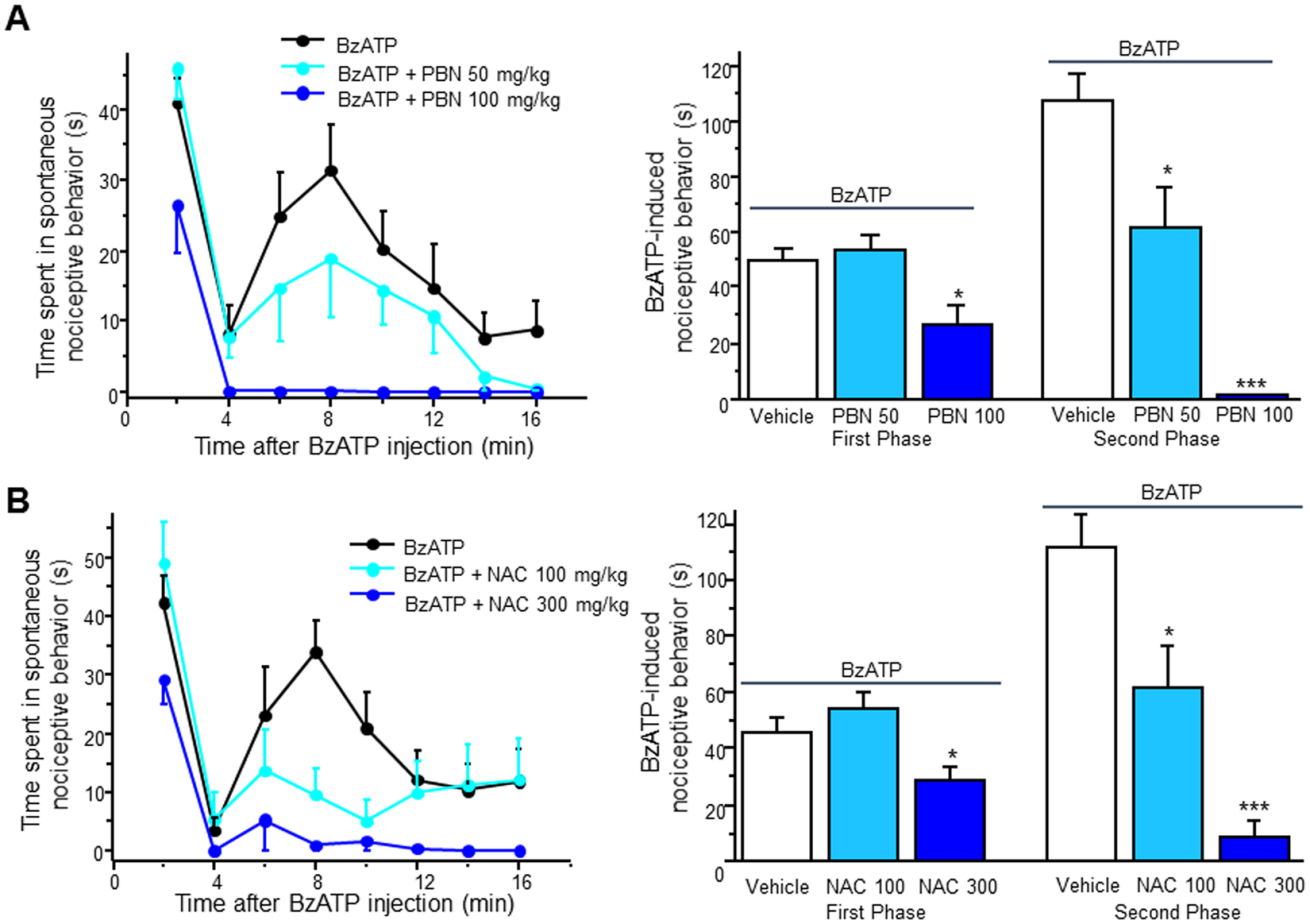
ROS scavengers attenuate BzATP-induced spontaneous nociceptive behavior. (**A**) Left panel: Time course of BzATP-induced spontaneous nociceptive behavior with and without PBN pre-treatment. Right panel: Total time spent in spontaneous nociceptive behavior. First phase includes time 0–4 min. Second phase includes 4–16 min. (**B**) Left panel: Time course of BzATP-induced spontaneous nociceptive behavior with and without NAC pre-treatment. Right panel: Total time spent in spontaneous nociceptive behavior. Values for both represent mean ± SEM, n = 6–8. *P < 0.05 and ***P < 0.001 when compared to BzATP by the one-way ANOVA.

To determine whether ROS play a role in the mechanisms by which P2X7R contributes to pain, we tested the effect of ROS scavengers on P2X7R-induced spontaneous nociception. Similar to our previous experiments, mice were pre-treated for 30 min with PBN (50–100 mg/kg, i.p.) or N-acetyl-L-cysteine (NAC, i.p. 100–300 mg/kg, i.p.) prior to a single intrathecal administration of BzATP. PBN significantly reduced the first response induced by BzATP at higher doses (Fig. 8A). Interestingly, PBN dose-dependently attenuated the secondary response induced by BzATP, and completely abolished the secondary response at the higher dose (Fig. 8A). To confirm our *in vivo* finding with PBN, we employed a different type of ROS blocker, NAC, a commercially available and well-established antioxidant that increases the amount of free radical scavengers intracellularly. Pre-treatment with NAC seemed to produce almost identical results to the PBN pre-treatment. NAC dose-dependently decreased the second phase of response, and completely blocked the secondary response at the higher dose (Fig. 8B). The higher dose of NAC was also able to reduce the BzATP-induced first response (Fig. 8B). Thus, our data suggest that ROS play a significant role in P2X7R-induced spontaneous nociceptive pain.

## Discussion

Our present study demonstrates that neuronal oxidative stress contributes to P2X7R-induced nociceptive behavior. P2X7R was directly activated in about 75% of cultured SCDH neurons, which subsequently increased ROS production through NADPH oxidase. Consistent with our *in vitro* results, P2X7R activation by BzATP increased ROS in the SCDH of mice, and oxidative DNA damage occurred in neurons. Moreover, BzATP-induced spontaneous nociceptive behavior was dependent on ROS increases. Together, our results establish a link among P2X7R activation in neurons, increases in ROS production, and nociceptive behavior.

It is well recognized that P2X7R activation leads to increased ROS production. Many studies have reported ROS increase in macrophages, microglia, and many other diverse cell types^17, 25, 26^. Moreover, P2X7R is expressed and functional in neurons^13, 15^. Thus, we hypothesized activation of P2X7R could also increase ROS production in SCDH neurons. Consistent with our hypothesis, our calcium imaging and live cell imaging experiments clearly show that BzATP directly activated neuronal P2X7R, which led to increases in ROS. Interestingly, ATP itself increased ROS production in neurons, but the specific P2X7R antagonist, A438079, only partially attenuated this increase. It is therefore possible other P2 purinoreceptors may be involved in ATP-induced ROS production. A recent report demonstrated ATP can induce ROS production through P2Y1 receptor activation in skeletal muscle cells^45^, and others have also reported ROS generation following P2Y1 receptor activation in astrocytes and neurons^46, 47^. Another study indicates that P2X4 receptor negatively regulates P2X7R-induced ROS production in macrophages^48^, thus suggesting P2 receptors may indeed regulate one another.

Previous reports have suggested that P2X7-induced ROS production is NOX2 dependent in microglial cells^37, 49^. Other studies have also shown inhibition with apocynin, which prevents the assembly of NOX^50^, attenuates P2X7R-mediated ROS increases, suggesting ROS production may indeed require NOX^36, 51^. Similarly, we found apocynin abolished BzATP-induced ROS production in SCDH neurons. Thus, our data indicate that P2X7R-mediated ROS production may be through NOX. It has been reported that Rac1, a Rho-GTPase family member, is a central activator of NOX1 and NOX2^52, 53^. A recent study also states that P2X7R-mediated NOX2 activation and subsequent ROS production were completely dependent on Rac1 in microglia^37^. Therefore, it is possible that P2X7R activation leads to Rac1 activation and subsequent NOX activation in SCDH neurons.

To confirm the ROS increases observed *in vitro* following P2X7R activation, we measured ROS production in the spinal cord of mice following an intrathecal injection of BzATP. As expected, ROS robustly increased in the L4-L5 SCDH of mice following P2X7R activation, an effect completely abolished by pre-treatment with either the ROS scavenger PBN or the specific P2X7R antagonist A438079. NADPH oxidase plays a dominant role in P2X7R-induced ROS^28^, and creates ROS through formation of superoxide^29^. Thus, measuring oxidative stress with DHE staining, a superoxide indicator, directly assesses ROS produced by P2X7R activation. To determine whether ROS production occurred in neurons, spinal cord slices were stained with the oxidative DNA damage marker, 8-oxo-dG. Similar to our DHE staining results, 8-oxo-dG increased substantially in the SCDH of mice. Interestingly, much of 8-oxo-dG colocalized with NeuN, a commonly used neuronal marker, thus suggesting that P2X7R is activated in neurons and leads to ROS production in the spinal cord. There is, however, the possibility that neuronal ROS may have increased in the spinal cord partially through an indirect mechanism, such as activation of P2X7R in microglia. Studies have shown a close interaction among neurons, microglia, and astrocytes in the CNS, particularly communicating through gliotransmitters such as ATP and glutamate^54^. P2X7R is also present in spinal microglia and astrocytes, and others have reported activation of astrocytic and microglial P2X7R activation can lead to neuronal damage, and even cell death^55, 56^. Moreover, whole cell recordings done on hippocampal tissue slices demonstrated that BzATP can immediately activate strong inward currents in microglia, suggesting microglial activation of P2X7R and subsequent downstream signaling can occur prior to Iba1 development^57^. We therefore do not rule out the possibility that neuronal ROS production in mice may be in part due to an indirect effect from P2X7R activation in other cell types in the CNS. However, our data have demonstrated there is no microglial or astrocytic activation in the spinal cord following acute intrathecal administration of BzATP. Additionally, in our *in vitro* studies, we have included NMDA and AMPA receptor inhibitors that prevent neuronal activation from glutamate release of neighboring cells. Thus, our data suggest that neuronal ROS production likely occurs independently of astrocytic and microglial activation under our conditions.

Many studies have shown that P2X7R is involved in neuropathic pain and inflammatory pain^58^. Mice lacking P2X7R show no hypersensitivity to mechanical and thermal stimulation^32^. Moreover, several studies have demonstrated that specific P2X7R antagonists attenuate or completely abolish hypersensitivity to thermal and mechanical stimulation in chronic neuropathic and inflammatory pain models^59–61^. While P2X7R activation has been more extensively studied in chronic pain models, some studies have also reported that P2X7R plays a role in acute inflammation^62, 63^. Here, we show BzATP induced robust spontaneous nociceptive behavior in mice through P2X7R activation, thus suggesting P2X7R may be involved in acute nociception.

ROS have been heavily implicated in many neurodegenerative diseases and are critically important in the development of pain^18, 64^. Moreover, one study has found that a specific type of ROS, superoxide, mediates inflammatory pain^20^. In our study, pre-treatment with both an ROS scavenger and an antioxidant significantly attenuated the second phase of nociceptive behavior, while higher doses partially reduced the first phase and completely abolished the second phase of response. Interestingly, previous studies have reported significant analgesia from both PBN and NAC in neuropathic and inflammatory pain models^23, 65–67^. While how ROS lead to acute pain is not well understood, others have also reported ROS-mediated acute nociceptive behavior. For example, NAC can attenuate nocifensive behavior induced by the formalin test^68^. Additionally, in a recent report, ROS production contributed to spontaneous nociceptive behavior induced by administration of bee venom, and administration of ROS scavengers and antioxidants significantly attenuated the behavior^69^. We therefore conclude that an increase in neuronal ROS production contributes to P2X7R-induced spontaneous nociceptive behavior, particularly in the second phase of response.

## Methods

### Animals

All experiments were done in accordance with the guidelines of the National Institutes of Health, the Committee for Research and Ethical Issues of IASP, and were approved by the Animal Care and Use Committee of Drexel University College of Medicine. Pregnant mice were purchased from Charles River (Wilmington, MA) or Taconic (Hudson, NY) and individually housed in standard cages and maintained on a 12-h light/dark cycle. CD1 and C57BL/6 neonatal mice were used for cell cultures and C57BL/6 adult mice were used for behavior experiments.

### Cell Culture

Primary cultures of spinal cord superficial dorsal horn neurons were prepared from neonatal (P0 or P1) mice as previously described^70^. Briefly, neonatal mice were decapitated after inducing hypothermia on ice. A laminectomy was performed and the spinal cord was carefully removed. The superficial dorsal horn was dissected with a surgical blade cut in approximately lamina III. The superficial dorsal horn strips were incubated for 30 min at 37 °C in HBSS (Life Technologies, Carlsbad, CA) (in mM: 137 NaCl, 5.4 KCl, 0.4 KH_2_PO_4_, 1 CaCl_2_, 0.5 MgCl_2_, 0.4 MgSO_4_, 4.2 NaHCO3, 0.3 Na_2_HPO_4_, and 5.6 glucose) containing papain (7.5 U/ml; Worthington Biochemical, Lakewood, NJ), rinsed three times with HBSS, and placed in culture medium containing Neurobasal A (Life Technologies), fetal calf serum (2%; Life Technologies), heat-inactivated horse serum (2%; Invitrogen), L-glutamax-1 (0.2 mM; Life Technologies), and B-27 (2%; Life Technologies). The strips were mechanically dissociated by gently triturating with a pipette. The resulting cells (88% neurons, stained with a neuron marker NeuN) were plated onto poly-D-lysine- and laminin-coated coverslips. Cells were maintained at 37 °C in a humidified atmosphere containing 5% CO_2_ for 2 to 4 days.

### Drugs

Adenosine 5′-triphosphate disodium salt hydrate (ATP), N-acetylcysteine (NAC), apocynin and N-tert-butyl-α-phenylnitrone (PBN) were purchased from Sigma Aldrich (St. Louis, MO). CNQX, D-AP5, 2′(3′)-O-(4-Benzoylbenzoyl)adenosine-5′-triphosphate tri(triethylammonium) salt (BzATP) and A438079 were purchased from Tocris (Bristol, UK). All compounds were dissolved in Milli-Q water or dimethyl sulfoxide (DMSO) as stock solutions and further diluted to final concentrations in 0.1% DMSO.

### Calcium imaging

Calcium imaging was performed using fura-2-based microfluorimetry and imaging analysis as we previously described^71^. Neurons were plated at 4 × 10^4^ cells/ml in 12 mm coverslips and allowed to grow for 1–2 days. Neurons were then loaded with 4 µM of fura-2AM (Life Technologies, Grand Island, NY) for 30 min at room temperature in HBSS, washed, and further incubated in normal bath solution (Tyrode’s) containing (in mM) 140 NaCl, 5 KCl, 2 CaCl_2_, 1 MgCl_2_, 10 HEPES, and 5.6 glucose (pH 7.4) for 20 min. Images were acquired at 3-sec intervals at room temperature (20–22 °C) using an Olympus inverted microscope equipped with a CCD camera (Hamamatsu ORCA-03G, Japan). The fluorescence images were recorded and analyzed using the software MetaFluor 7.7.9 (Molecular Devices). The fluorescence ratio was determined as the fluorescence intensities excited at 340 and 380 nm with background subtraction. Neurons with a Ca^2+^ response ratio higher than 0.2 were accepted for analysis. Only one recording was made from each coverslip.

### Live Cell Confocal Imaging for ROS measurement

Neurons were plated at 4 × 10^4^ cells/ml in 12 mm glass bottom dishes 24–48 hrs prior to imaging. Cells were loaded with 5 µM CellROX Green (Life Technologies, Carlsbad, CA) at 37 °C for 30 min in Tyrode’s solution according to manufacturer’s instructions. Cells were then washed twice with Tyrode’s solution, incubated in D-AP5 (30 µM) and CNQX (10 µM) for 10 min and fluorescence images were captured using the Olympus FLUOVIEW FV1000 confocal microscope equipped with a 60x oil-immersion objective. Time lapse imaging was performed in CellROX loaded neurons. Images were acquired at 2–4 min intervals using a 488-nm laser line for excitation, and emission through a 520-nm window. The increases in fluorescence were quantified using Image J by analyzing individual cells for mean intensity. Values were normalized to intensity at time 0 of control samples.

### Detection of ROS generation in spinal cord tissue

C57BL/6 mice were pre-treated with an intraperitoneal injection of PBN or saline for 30 min, then given an intrathecal injection of BzATP (30 nmol). After 10 min, animals were deeply anesthetized using isoflurane. The lumbar spinal cord (L4–L5) was collected then flash frozen immediately in Tissue-Tek O.C.T. Compound (Sakura Finetek, VWR, Radnor, PA) on a dry ice bath containing 70% ethanol. Spinal cord sections were cut into 30 µM thick slices and placed on a glass Superfrost slide, then heated for 30 min at 45 °C. 1 µM dihydroethidium (DHE) (Life Techonologies, Carlsbad, CA), an ROS indicator, was then applied to each slice, and the slides were covered with coverslips. Glass slides were then incubated in a dark, humidified chamber at 37 °C for 30 min. Fluorescent images of the superficial dorsal horn were captured using the Olympus FLUOVIEW FV1000 confocal microscope equipped with a 30x oil-immersion objective using a 515-nm laser line for excitation, and emission through a 600-nm window. Imaging capture parameters, such as gain, offset, HV, and laser intensity, were kept constant during the acquisition of images for valid comparisons of DHE intensity within a study. Quantitative analysis was performed by using Image J to measure mean fluorescence intensity from at least 3 randomly chosen spinal cord slices from each animal.

### Immunofluorescent staining

C57BL/6 mice were pre-treated with an intraperitoneal injection of PBN or saline for 30 min, then given an intrathecal injection of BzATP (30 nmol). After 10 min, animals were deeply anesthetized using isoflurane and perfused intracardially with saline followed by 4% cold buffered paraformaldehyde (PFA)/0.1 M phosphate buffer (PB) solution. The lumbar spinal cord (L4–L5) was removed then post-fixed overnight in 4% PFA/0.1M PB, followed by cryoprotection in 30% sucrose in 0.1M PB for 2 days. Spinal cords were embedded in Tissue-Tek O.C.T. Compound (Sakura Finetek, VWR, Radnor, PA), and sections were cut into 30 µM thick slices and blocked with PBS containing 5% normal goat serum (NGS) and 0.3% Triton-X 100 for 1 h. Primary antibodies for microglia (Iba1 1:100, BD Biosciences, San Jose, CA), astrocytes (GFAP 1:100, BD Biosciences), neurons (NeuN 1:100, Millipore, Billerica, MA), and/or oxidative DNA damage marker 8-oxo-dG (1:500, Trevigen, Gaithersburg, MD) were incubated overnight at 4 °C in blocking solution. After 3 washes in PBS, slices were incubated with secondary antibodies (Alexa Fluor, ThermoFisher, Waltham, MA) for 1 h in blocking solution. Spinal cord slices were then mounted on glass slides and coverslips were applied using mounting media (Southern Biotech, Birmingham, AL). Images of the superficial dorsal horn were captured using the Olympus FLUOVIEW FV1000 confocal microscope equipped with a 30x oil-immersion objective. Imaging capture parameters, such as gain, offset, HV, and laser intensity, were kept constant during the acquisition of images for valid comparisons of Iba1, GFAP, and 8-oxo-dG intensity within a study. Quantitative analysis was performed by using Image J to measure mean fluorescence intensity from at least 3 randomly chosen spinal cord slices from each animal. Intensity of the spinal cord lamina I, II and III were measured for Iba1 and 8-oxo-dG intensity, while lamina I was measured for GFAP, where astrocytes were clearly observed.

### Behavior Studies

Behavioral testing was performed with the investigator blind to treatments using 7–9-week-old C57BL/6 male mice. Animals were placed in clean plastic mouse cage for at least 1 h before experiment. BzATP-induced nociceptive behavior was elicited by intrathecal injection of BzATP (30 nmol) and assayed by recording the total time spent in spontaneous nociceptive behavior (caudally oriented licking of the flanks, tail, and hind paws) in 2 min intervals for 16 min.

### Statistical Analysis

Data are expressed as mean ± SEM. Treatment effects were statistically analyzed with a one-way analysis of variance (ANOVA). When ANOVA showed a significant difference, pair wise comparisons between means were performed by the post hoc Bonferroni method. Paired or two-sample Student’s *t* tests were used when comparisons were restricted to two means. Error probabilities of *P* < 0.05 were considered statistically significant. The statistical software Origin 8.1 was used to perform all statistical analyses.

## Electronic supplementary material

Supplementary info
