# Supplementary material for: Neuronal P2X7 receptor-induced reactive oxygen species production contributes to nociceptive behavior in mice
Source: Sci Rep. 2017 Jun 14;7:3539. doi: 10.1038/s41598-017-03813-7 (PMC5471238; doi:10.1038/s41598-017-03813-7)
Supplement: Supplementary file 1 — Supplementary info [file 41598_2017_3813_MOESM1_ESM.pdf]

Neuronal P2X7 receptor-induced reactive oxygen species production contributes to nociceptive behavior in mice

Authors: Frances M Munoz, Ruby Gao, Yuzhen Tian, Brian A. Henstenburg, James E Barrett, and Huijuan Hu

Supplementary information

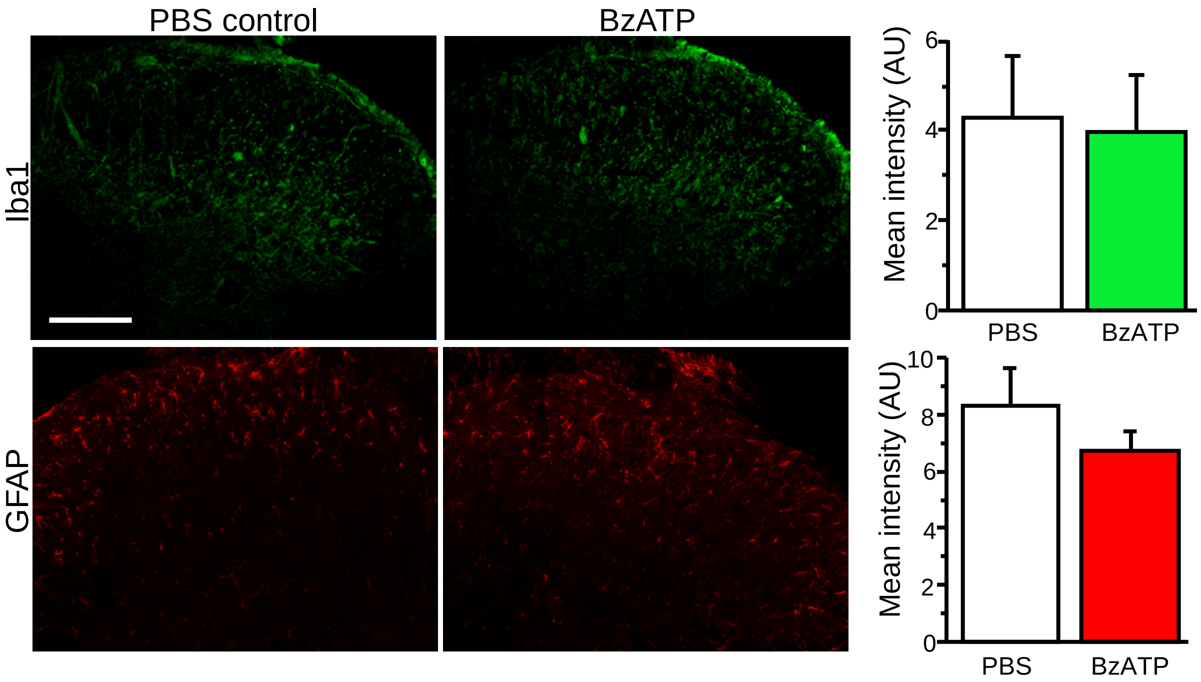

Supp Figure 1. Acute BzATP intrathecal administration does not activate microglia or astrocytes in the spinal cord dorsal horn. C57BL/6 mice were given an intrathecal injection of PBS (control) or BzATP (30 nmol). Left panel: Confocal images of spinal cord dorsal horns stained with microglial activation marker Iba1 (green) or astrocyte activation marker GFAP (red). Right panel: Summary of mean intensity of Iba1 (top) and GFAP (bottom) induced by BzATP. No statistical significance was achieved by the unpaired Student's t test. Values represent mean ± SEM, n=3-4. Scale bar 100 µm.
